# Supplementary material for: The Effect of Capping Agents on Gold Nanostar Stability, Functionalization, and Colorimetric Biosensing Capability
Source: Nanomaterials (Basel). 2022 Jul 19;12(14):2470. doi: 10.3390/nano12142470 (PMC9319646; doi:10.3390/nano12142470)
Supplement: Supplementary file 1 [file nanomaterials-12-02470-s001.zip › nanomaterials-1795964-supplementary.pdf]

nanomaterials

Supplementary Materials: The Effect of Capping Agents on Gold Nanostar Stability, Functionalization, and Colorimetric Biosensing Capability

Tozivepi Aaron Munyayi *, Barend Christiaan Vorster * and Danielle Wingrove Mulder

Particle size distribution of the AuNS bioconjugates

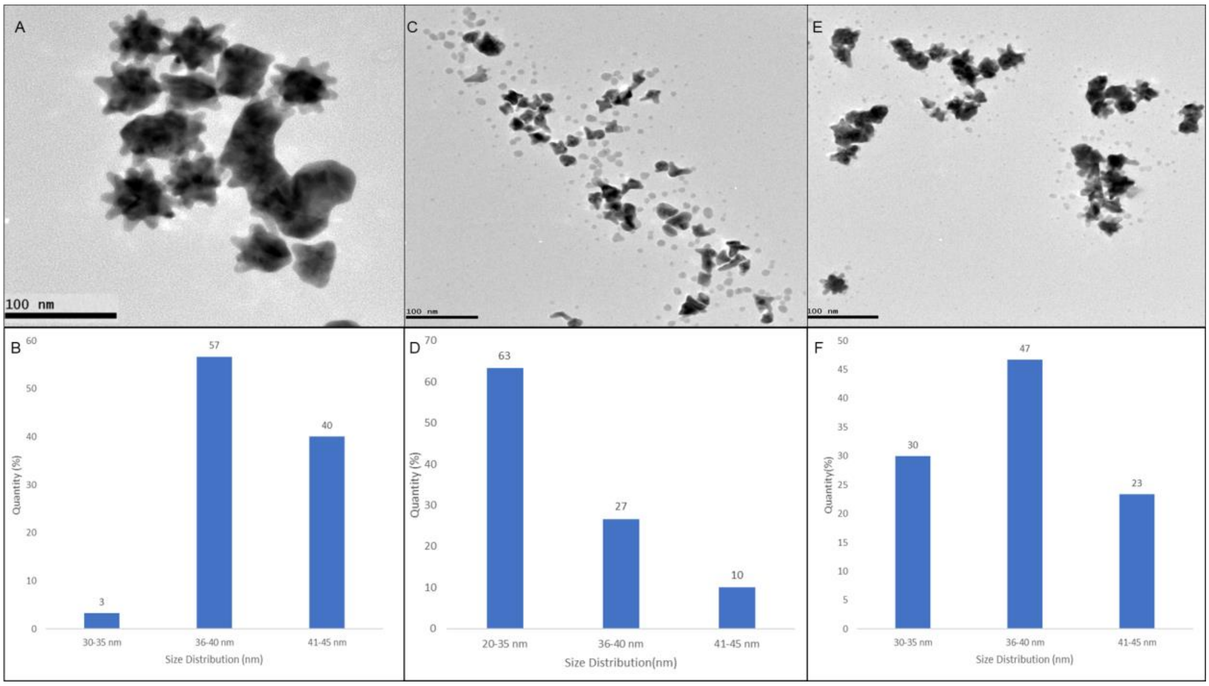

Figure S1. TEM image comparison of bioconjugates and size distribution: (**A**–**B**) AuNS-PVP-GOx; (**C**–**D**) AuNS-PEO-GOx; and (**E**–**F**) AuNS-PEG-GOx.

Of the 100 nanostars analyzed for each sample, a greater percent were 12-armed nanostars, whereas the remainder were more spherical with a few short protruding arms.

Confirming AuNS auto degeneration in the presence of GOx

AuNS-GOx showed morphological change within 30 min of synthesis, as confirmed by 5 min intermittent spectrophotometric readings and suspension color change visible to the naked eye (see Figure S2A). The TEM image shows the structural and morphological changes in the AuNS-GOx bioconjugate.

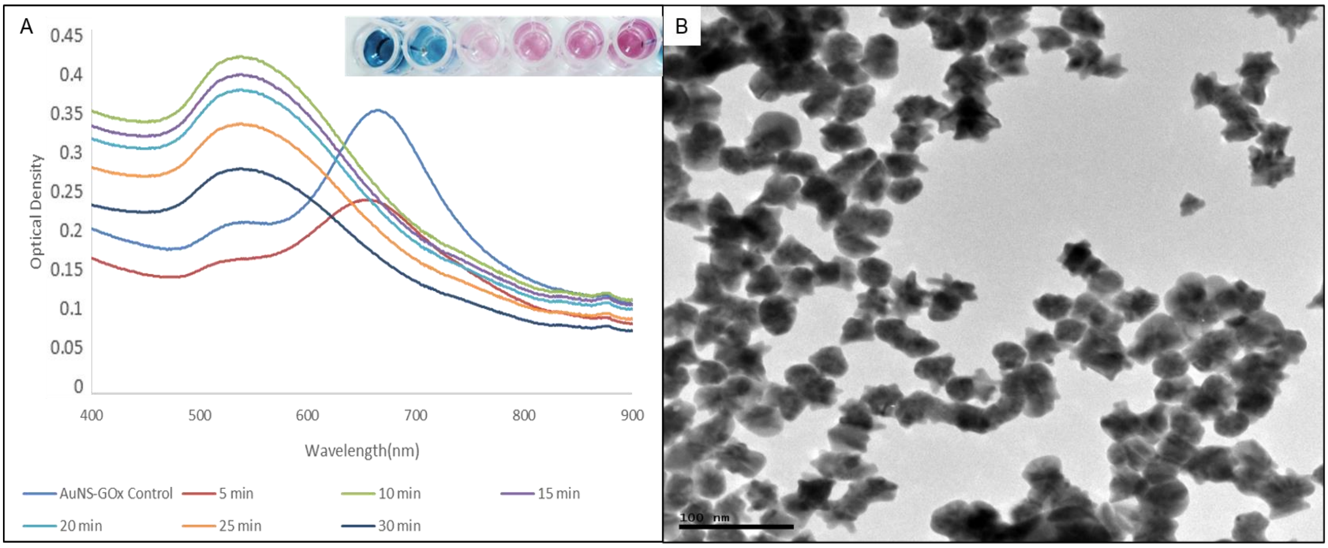

Figure S2. Morphological changes of AuNS-GOx bioconjugate: (**A**) UV-Vis spectra and (**B**) TEM image.

Confirming AuNS sensitivity to H_2O_2 and colourimetric signal generation

A pilot colorimetric assay study for the sensitivity of capped AuNSs to various H_2O_2 concentrations was assessed in Tris buffer, as presented in Figure S3. The color changes observed ranged from deep purple to light purple for AuNS, deep orange to light orange for AuNS-PVP, lime green to orange for AuNS-PEO, and pale -yellow shades to light green for AuNS-PEG. It was noted that capping agents resulted in different colorimetric readouts, and in all the spectra, the longitudinal peak of AuNS was absent, leaving behind the transverse peak. A hypsochromic effect (blue shift) was noted from the UV-Vis spectra relative to the control. The capped AuNS produced excellent color hues visible to the naked eye. There were no color changes observed when various reaction mixture components were omitted.

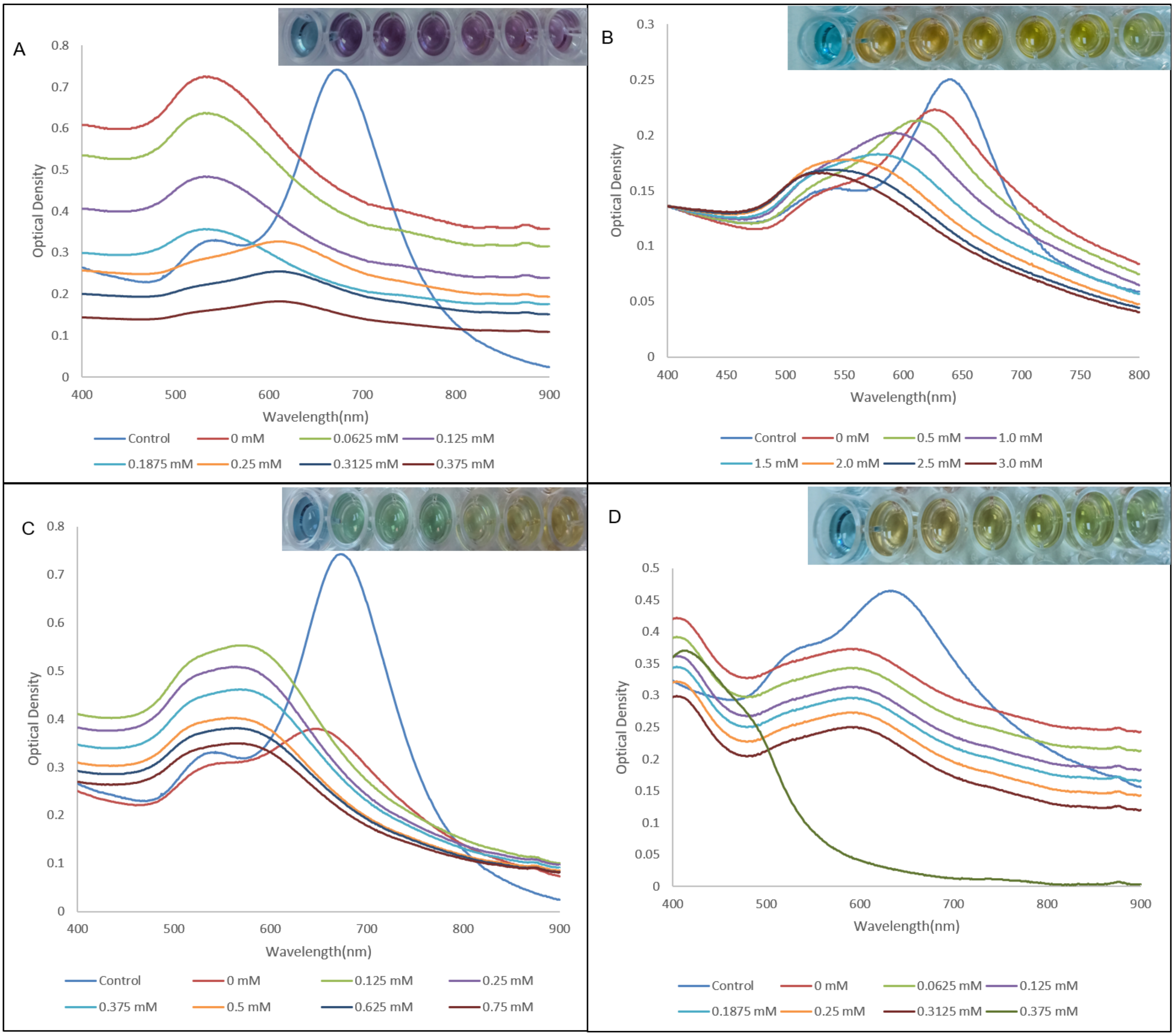

Figure S3. Hydrogen- peroxide- sensing efficacy UV-Vis spectra: (**A**) AuNS; (**B**) AuNS-PVP; (**C**) AuNS-PEO; and (**D**) AuNS-PEG.

It was also found that the spiked GOx procedure produced very similar colors, as confirmed by the inserts (as seen in Figure S4) and the UV-Vis spectra, which showed poor separation. The enzyme orientation on the nanoparticle surface was not controlled. This passive (or physisorption) process should be attentively governed for reproducible conjugates.

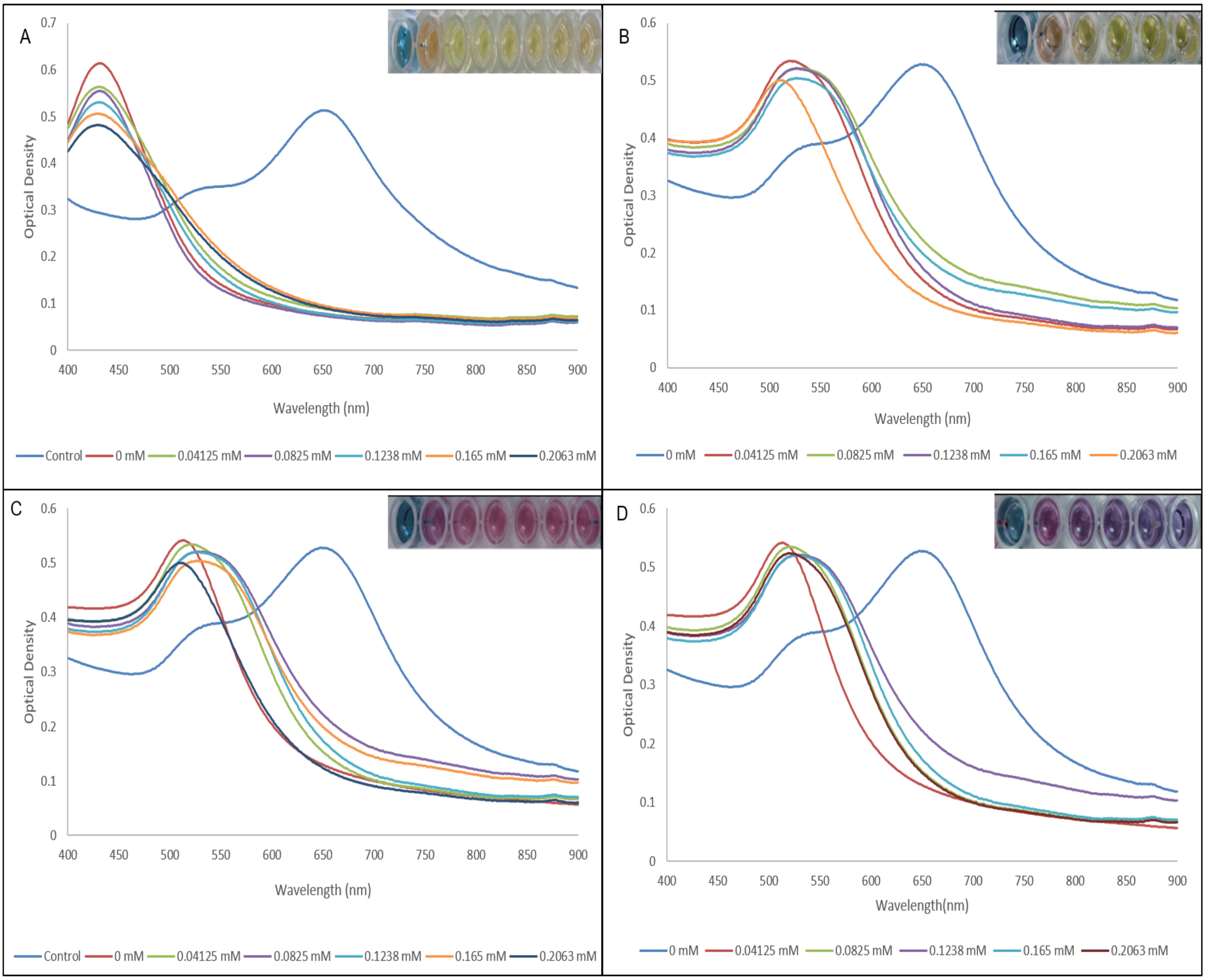

Figure S4. UV-Vis spectra and colorimetric readouts for Gox-spiked AuNSs: (**A**) AuNS; (**B**) AuNS-PVP; (**C**) AuNS-PEO; and (**D**) AuNS-PEG.
